# Pituitary Stalk Interruption Syndrome: Diagnostic Delay and Sensitivity of the Auxological Criteria of the Growth Hormone Research Society

**DOI:** 10.1371/journal.pone.0016367

**Published:** 2011-01-27

**Authors:** Géraldine Gascoin-Lachambre, Raja Brauner, Laetitia Duche, Martin Chalumeau

**Affiliations:** 1 Université Paris Descartes, AP-HP, Hôpital Bicêtre, Unité d'Endocrinologie Pédiatrique, Le Kremlin Bicêtre, France; 2 Université Paris Descartes, AP-HP, Groupe Hospitalier Cochin-Saint-Vincent-de-Paul, Service de Médecine Néonatale de Port-Royal, Paris, France; 3 Université Paris Descartes, AP-HP, Hôpital Necker Enfants Malades, Service de Pédiatrie Générale, Paris, France; 4 Inserm U953, Epidemiological Research Unit on Perinatal Health and Women's and Children's Health, Hôpital Saint-Vincent-de-Paul, Paris, France; Stanford University Medical Center, United States of America

## Abstract

**Objectives:**

To study the diagnostic delay for pituitary stalk interruption syndrome (**PSIS**) with growth hormone deficiency (**GHD**) and the sensitivity of the auxological criteria of the Growth Hormone Research Society (**GHRS**) consensus guidelines.

**Methods:**

A single-center retrospective case-cohort study covering records from January 2000 through December 2007 evaluated the performance of each GHRS auxological criterion for patients with GHD and PSIS. Diagnostic delay was calculated as the difference between the age at which the earliest GHRS criterion could have been observed and the age at diagnosis of PSIS with GHD. A diagnostic delay exceeding one year was defined as late diagnosis.

**Results:**

The study included 21 patients, 16 (76%) of whom had isolated GHD and 5 (24%) multiple pituitary hormone deficiencies. The median age at diagnosis was 3.6 years (interquartile range, **IQR**, 2.6–5.5). The median diagnostic delay was 2.3 years (range 0–12.6; IQR 1.5–3.6), with late diagnosis for 17 patients (81%). Height more than 1.5 SDS below target height was the most effective criterion: 90% of the patients met the criterion before diagnosis at a median age of 1 year, and it was the first criterion to be fulfilled for 84%.

**Conclusion:**

In our cohort, the delay for diagnosis of PSIS with GHD was long and could have been reduced by using the GHRS criteria, in particular, height more than 1.5 SDS below the target height. The specificity of such a strategy needs to be tested in healthy populations.

## Introduction

Growth hormone (**GH**) deficiency (**GHD**) can be congenital or acquired. The incidence of congenital GHD has been assessed at from 1/4000 to 1/10 000 [1], [2], [3], [4]. The pituitary stalk interruption syndrome (**PSIS**) is a sign of congenital and permanent GHD [5], [6], [7]. It is diagnosed by magnetic resonance imaging (MRI) and includes the absence of both a visible pituitary stalk and normal posterior lobe hyperintense signals in the sella turcica, together with the presence of a hyperintense nodule in the region of the infundibular recess of the third ventricle. Familial forms of PSIS and associated malformations suggest that its origin is antenatal [8]. It is important to diagnose GHD and start treatment as soon as possible because this deficiency is associated with excess mortality and substantial morbidity [9], [10]. Moreover, because insufficient height at the onset of puberty leads to short final height, early diagnosis and treatment of GHD are necessary to allow catch-up growth to optimal height before puberty [11]. Signs of congenital GHD in neonates include hypoglycemia, prolonged jaundice, and microphallus [1], [8], [12], [13]. In older children, the diagnosis is based on short stature or growth failure. Height for age is the most common criterion for referral for GH evaluation [14]. However, the mean ages reported for diagnosis of symptomatic PSIS in various studies range from 4 to 9 years and suggest important diagnostic delay [5], [7], [12], [13], [15].

In 2000, the GH Research Society (**GHRS**) published guidelines based on height for age but also five other auxological criteria (see below), to ensure that children and adolescents with GHD are appropriately identified and treated [16]. A survey has shown that these criteria are not currently applied, probably because the concomitant use of six auxological criteria might be difficult in day-to-day routine practice [14]. Moreover the performance (notably sensitivity for early diagnosis) of these guidelines has never been tested.

The objective of this study was therefore to study the diagnostic delay for PSIS with GHD and the sensitivity of the auxological criteria of the GHRS to identify the most useful ones and simplify their routine use.

## Results

### Characteristics of the population

During the study period, 67 patients seen for growth failure had PSIS and/or GHD: 38 (57%) had GHD with a normal MRI or an isolated hypoplastic anterior pituitary gland, 2 (3%) had GHD and PSIS but had been adopted, six (9%) patients had GHD and PSIS diagnosed in the neonatal period. The study thus included 21 (31%) patients with GHD and PSIS (Table 1), 76% of them boys. One patient was born preterm, and nine were delivered by cesareans (43%) (confidence interval, **CI** = 22–64), including three in breech presentation. One patient had midline abnormalities, including bilateral optic nerve hypoplasia.

**Table 1:** Patient characteristics.

	Isolated GHD (n = 16)		MPD (n = 5)		TOTAL (n = 21)	
**Neonatal symptoms**	n'	Percentage	n'	Percentage	n'	Percentage
Breech delivery	2	12.5% (CI 0–29)	1	20% (CI 0–55)	3	14% (CI 0–29)
Cesarean delivery	5	31% (CI 8–54)	4	80% (CI 45–100)	9	43% (CI 22–64)
**At diagnosis**		Median (range)		Median (range)		Median (range)
Age (yr)	16	3.2 (1; 13.6) (IQR 2.6; 4.9)	5	5.1 (1; 10.5) (IQR 5; 5.6)	21	3.6 (1; 13.6) (IQR 2.6; 5.5)
Bone age (yr)	12	1.5 (0.5; 9.5) (IQR 1.2; 2.3)	4	2.2 (0.5; 4) (IQR 1.6; 2.9)	16	1.7 (0.5; 9.5) (IQR 1.2; 2.5)
Bone age delay (yr)	12	1.3 (0.5; 4.1) (IQR 1; 1.7)	4	2.8 (0.5; 6.4) (IQR 2; 3.9)	16	1.4 (0.5; 6.4) (IQR 1; 2.6)
Target height (SDS)	16	−0.2 (−1.6; 1.5) (IQR –0.7; 0.3)	5	−0.3 (−1.5; 0.6) (IQR –0.6; 0.4)	21	−0.3 (−1.6; 1.5) (IQR –0.6; 0.4)
Height (SDS)	16	−2.7 (−4.3; −1.3) (IQR –3.7; −2.3)	5	−2.2 (−2.4; −2) (IQR –2.2; −2)	21	−2.5 (−4.3; −1.3) (IQR –3.5; −2)
Height velocity (SDS)	16	−3 (−4.1; 0.3) (IQR –3.3; −1.6)	5	−3.3 (−4.2; 0) (IQR –3.4; −3.2)	21	−3.1 (−4.2; 0.3) (IQR –3.4; −1.6)
Weight (SDS)	16	−2.5 (−4; −0.4) (IQR –3; −1.9)	5	−0.7 (−1.3; 1.1) (IQR –1.2; −0.3)	21	−2.4 (−4; 1.1) (IQR –2.8; −1)
BMI (SDS)	16	−0.9 (−3.7; 2.2) (IQR –1.5; 0.2)	5	1.3 (−0.2; 4) (IQR –0.1; 1.7)	21	−0.23 (−3.7; 4) (IQR –1.1; 0.5)
GH peak (ng/mL)	16	3.2 (1.5; 23) (IQR 2; 6.7)	5	2.1 (0.5; 4.1) (IQR 0.9; 3.1)	21	3 (0.5; 23) (IQR 2; 5.5)
IGF-1 (ZS)	16	−2.9 (−5.1; −2) (IQR –4; −2.4)	5	−4.8 (−5; −4.1) (IQR –4.9; −4.4)	21	−3.1 (−5; −2) (IQR –4.4; −2.7)

CI: confidence interval 95%.

IQR: interquartile range.

GHD: growth hormone deficiency.

MPD: multiple pituitary deficiencies.

SDS: standard deviation score.

ZS: Z-score.

Median age at diagnosis was 3.6 years (range 1–13.6; interquartile range **IQR**: 2.6–5.5), and all patients were prepubertal (Table 1). Sixteen patients (76%) (95%) (CI 58–94) had isolated GHD and five (24%) (CI 6–42) had multiple pituitary deficiencies (**MPD**) with thyroid stimulating hormone deficiency in four and adrenocorticotrophin deficiency in two. The median height was −2.5 SDS (range −4.3; −1.3) (IQR −3.5; −2) and median BMI −0.23 SDS (range −3.7; 4) (IQR −1.1; 0.5). The median height velocity was −3.1 SDS (range −4.2; 0.3) (IQR −3.4; −1.6).

### Medical and growth history

Nine families (43%) (CI 22–64) first consulted a private-practice pediatrician about growth failure, and 12 families an outpatient pediatric department (57%) (CI 36–78). One family sought care directly from our team. Eleven patients (52%) (CI 31–73) had undergone laboratory testing for growth retardation before consulting our team, two had had a GH stimulation test, and 3 had had serum IGF-1 measured. Both GH stimulation tests were normal, and serum IGF-1 was less than –2 SDS, but no further diagnostic procedures were performed to rule out GHD. The patient with bilateral optic nerve hypoplasia had had neonatal hypoglycemia and microphallus but was not evaluated for GH secretion until the age of one year, and then for growth failure. His pediatrician had ordered an MRI at 2 months of age because his eyes were not yet following objects. At 5 months of age, his growth rate started to decrease and at one year of age, he was addressed to our department for growth retardation. The PSIS diagnosis was based on the MRI performed at 2 months of age.

No episodes of severe hypoglycemia or adrenal crisis were observed before diagnosis, and no child had any neurological deficiency.

### Performance of GHRS criteria

Table 2 summarizes the performance of each GHRS criterion. The criterion of height more than 2 SDS below the mean + height velocity over 1 year more than 1 SDS below the mean for chronological age had a frequency at final diagnosis of 100%. Height more than 1.5 SDS below the target height was the most effective criterion: 90% of the patients had met the criterion before diagnosis, at a median age of 1 year (range 0; 9) (IQR 0.5; 1.8), and it was the first criterion to be met for 84% of the patients. Its use could have reduced diagnostic delay by 2.1 years (range 0; 12.6) (IQR 1.5; 2.9). The combined use of these two criteria, height more than 2 SDS below the mean + height velocity over 1 year more than 1 SDS below the mean for chronological age and height more than 1.5 SDS below the target height, might also have reduced diagnostic delay by 2.1 years (range 0; 12.6) (IQR 1.5; 3) for a median age at first validation of one of these criteria, that is, the first visit at which a doctor could have determined that the criterion had been met, was 1 year (range 0; 4.7) (IQR 0.6; 2).

**Table 2:** Individual analysis of auxological GHRS criteria.

	Height <−3 SDS	Height <−1,5 SDS below the target height	Height <−2 SDS and height velocity <−1 SDS*	Height <−2 SDS and height diminution >0,5 SDS**	Normal height and height velocity <−2 SDS*	Normal height and height velocity <−1,5 SDS***	At least one of the 6 criterion
Criterion completed at diagnosis n (%) (CI)	11 (52%) (31–73)	19 (90%) (77–100)	21 (100%) (100–100)	11 (52%) (31–73)	5 (24%) (6–42)	4 (19%) (2–36)	
Age at criterion completion (yr) median (range) (IQR)	1 (0,6; 10) (0.7; 2.2)	1 (0; 9) (0.5; 1.8)	2 (1; 9) (1; 3.9)	3 (2; 6) (3; 4.3)	3 (2; 6) (3; 4)	3 (2; 4) (2.7; 3.2)	1 (0; 4) (0.6; 2)
Number of patients who completed the criterion first n (%) (CI)	2 (18%) (0–41)	16 (84%) (67–100)	4 (19%) (2–36)	0 (0%) (0–0)	2 (40%) (3–83)	1 (25%) (0–67)	
Potential reduction of diagnostic delay among the patients who completed the criterion (yr) median (range) (IQR)	2 (0; 6.8) (0.1; 3.3)	2.1 (0; 12.6) (1.5; 2.9)	1.5 (0; 9.6) (0; 3)	0 (0; 1.5) (0; 0.3)	2 (0.6; 4.5) (0.7; 2.1)	2.7 (0.5; 6.5) (1.6; 4.2)	
Potential reduction of diagnostic delay among all patients (yr) median (range) (IQR)	0 (0; 6.8) (0; 2)	2 (0; 12.6) (0.6, 2.8)	1.5 (0; 9.6) (0; 3)	0 (0; 1.5) (0; 0)	0 (0; 4.5) (0; 0)	0 (0; 6.5) (0; 0)	2.3 (0; 12.6) (1.5; 3.6)

*over 1 year.

**over 1 year in children older than 2 years of age.

***over 2 years.

CI: confidence interval 95%.

IQR: interquartile range.

SDS: standard deviation score.

### Late Diagnosis

Median age at diagnosis was 3.6 years (range 1; 13.6) (IQR 2.6; 5.5). Median age when the auxological criterion was met was 1 year (range 0; 4) (IQR 0.6; 2). The median diagnostic delay was 2.3 years (range 0; 12.6) (IQR 1.5; 3.6), with late diagnosis in 17 patients (81%).

## Discussion

### Main results

We analyzed the diagnostic delay and sensitivity for the GHRS auxological criteria in the largest reported cohort of children seen for PSIS with GHD since the publication of these criteria. We studied the GHRS guidelines rather than other rules, such as the Dutch consensus guidelines or the UK guidelines, because it has been already demonstrated that both of these European guidelines lack specificity [17], [18], [19] or sensitivity [17], [19]. A Dutch team recently proposed another algorithm to identify children with short stature who require a diagnostic work-up, but this algorithm did not target PSIS with GHD as a key diagnosis [20]. We chose to study patients with GHD and PSIS because they comprise a homogeneous population with a permanent GHD, and because the real clinical significance of GHD without PSIS (diagnosed by a low GH response after 2 pharmacological stimulation tests and normal MRI) is a matter of debate today [21].

In all, 71% of patients had a diagnostic delay greater than 1 year. Correct application of the GHRS auxological criteria could have allowed diagnosis of these patients and the beginning of their treatment 2 years earlier. Of the GHRS criteria, the most effective for early and frequent diagnosis was height more than 1.5 SDS below the target height and the criterion met by all patients was height more than 2 SDS below the mean + height velocity over 1 year more than 1 SDS below the mean for chronological age. Height velocity and distance to target height have already been described by other teams as effective markers for detecting other growth disorders, such as Turner's syndrome, GHD and celiac disease [17], [22], [23].

Distance to target height and height velocity are still underused in routine practice [14]. Interestingly, height velocity is not included in the UK consensus guideline [19], [24], [25] nor as a growth monitoring indicator in the national French pediatric health notebook. It is not provided by the World Health Organization (WHO) growth charts after 24 months [19], not included in any study evaluating the effectiveness of height-screening programs [25], and was used by fewer than 50% of European pediatric endocrinologists in a 2002 survey [14].

The specificity of each of the best criteria identified by our study (height more than 1.5 SDS below the target height, as well as height more than 2 SDS below the mean + height velocity over 1 year more than 1 SDS below the mean for chronological age) could not be determined but can be compared indirectly with those of other studies [17], [22], [23]. The specificity of the Dutch guidelines for short stature was tested on a longitudinal growth data of 870 children born in a geographical area of the Netherlands [18]. Of the six criteria of the Dutch guidelines, the criteria of height more than −1.3 SDS below the mean and of height more than −1.3 SDS below the target height, which are close to one of our best criteria, had a specificity of 94%.

Although it may be somewhat difficult to use all GHRS criteria in routine practice to detect growth anomalies, our results for patients with GHD and PSIS as well as results from a larger population [22] indicate that distance to target height should be used routinely as a warning sign for growth anomalies to select the patients who require further investigation. It should replace height for age which is relatively insensitive for the detection of clinically relevant growth disorders [19].

Our work shows that GH peak is not enough to rule out a diagnosis of GHD. Indeed, GHD had been ruled out for 2 (10%) of the 21 patients included during their medical care because of normal GH peaks, despite serum IGF-1 less than –2 SDS. This observation supports the current modification of the use of GH provocative tests in the evaluation of GHD [21], [26], [27], [28]. Indeed, they are expensive, labor intensive, occasionally risky, and their results not very reproducible [28], [29], [30]. Their use has declined over the past two decades. Serum IGF-1, together with the growth rate, provides high quality diagnoses that are practical, simple and very accurate [30]. Patients suspected for GHD, with a BMI between –2 and +2 SDS, with very low IGF-1 levels should skip GH provocative tests and should be prescribed a MRI [29], [30].

### Study limitations

We used the national growth charts included in the French health notebook, developed in 1979 [31]. In 2006, the WHO multicentre growth reference study published growth charts for healthy breastfed infants living in good hygiene conditions [32]. The comparison of the anthropometric measurements of French children with the new WHO growth standards showed similarities for the neonatal measurements but differed substantially thereafter, with French measurements (height, weight and BMI) lower from 1 to 6 months and French height lower but BMI higher from 6 months to 5 years old [33]. The GHRS consensus guidelines do not make it clear which growth charts should be used. Testing the sensitivity of GHRS criteria by using WHO growth charts is thus probably necessary. Our study was limited to a single center, a design that can result in recruitment bias. The presence of such a bias is supported by the mean age at diagnosis of symptomatic PSIS in our cohort, 3.6 years, compared to those reported in the literature, 4 to 9 years [5], [7], [12], [13], [15], [21]. It is thus possible that diagnostic delays are greater in the general population and that application of the GHRS criteria would reduce diagnostic delays still more than it would have in our patients.

Adoption and uncertain paternity are common, limiting utility of the “target height” criterion. That is the reason why it may be useful to consider the use of a combination of our two best criteria: height more than 1.5 SDS below the target height and height more than 2 SDS below the mean + height velocity over 1 year more than 1 SDS below the mean for chronological age.

### Unexpected findings

We were surprised by the high proportion (14%) of breech presentation vs 4% in the general population in France [34], and by the high proportion of cesareans (43%) vs 25% in the general population in France [35]. Of patients with MPD, 20% were born in breech presentation, and 80% (including all patients with thyrotrophic insufficiency) were born by cesarean delivery. If we incorporate in the analysis the six excluded patients with PSIS diagnosed during the neonatal period, 22% of patients had breech presentations and 56% cesarean births, for all six were born by cesarean deliveries, three in breech presentation. We were not able to identify a selection bias that could explain this unexpected finding. The frequency of breech presentation and cesarean delivery for GHD patients in the literature varies respectively from 7 to 60% and 30 to 40% [36], [37], [38]. TSH and/or ACTH deficiency and/or GHD may play a role in labor or fetal mobility and lead to breech presentation and/or cesarean delivery. Although we certainly do not recommend a pituitary MRI for all newborns by cesarean delivery or with breech presentations, clinicians should be aware of this finding in determining which newborns with hypoglycemia require a diagnostic workup for GHD.

### Perspectives

Screening rules based on growth monitoring are currently a topic of debate [18], [19], [20]. Evidence-based strategies must be tested, both for their sensitivity for early diagnosis in case-cohort series of given target diseases (e.g., GHD, celiac disease, and Turner's syndrome) and for their specificity in healthy populations [23], [39]. The introduction of some of the GHRS criteria (especially height more than 1.5 SDS below the target height and height more than 2 SDS below the mean + height velocity over 1 year more than 1 SDS below the mean for chronological age) would probably be helpful for the early diagnosis of the target disease here, PSIS with GHD. However, the precise specificity of these criteria and their performance for the early diagnosis of other target diseases involving growth monitoring must be tested.

## Methods

### Study design

This single-center retrospective case-cohort study included all patients seen for PSIS with GHD by a senior pediatric endocrinologist (R Brauner) from January 2000 to December 2007. During the study period, the local routine protocol called for the systematic prescription of GH stimulation tests for all patients seen for growth failure and for systematic MRI of the hypothalamic-pituitary area of those with GHD (as defined below). All patients whose computerized hospital chart or discharge codes contained the words “growth hormone deficiency” and “pituitary stalk interruption syndrome” were considered for inclusion. The Institutional Review Committee (Comité de Protection des Personnes Ile de France III) stated that “this research was found to conform to generally accepted scientific principles and research ethical standards and to be in conformity with the laws and regulations of France, where the research experiment was performed.” Written informed consent of the patients or their parents was not judged necessary for this kind of retrospective study. The data of some of the patients included in the present were previously used for other purposes [26], [30].

### Inclusion criteria

We included all patients seen consecutively for GHD and PSIS. GHD was diagnosed by a GH peak of 10 ng/mL or less or 20 mIU/L or less after two pharmacological stimulation tests or a very low level of insulin-like growth factor (IGF)-1 (less than −2 standard deviation scores (**SDS**)) [40]. PSIS was diagnosed by MRI, according to the criteria described above. Patients with GHD but with a normal MRI or an isolated hypoplastic anterior pituitary gland were excluded, as well as adopted patients (because perinatal history and parental heights were not available). Patients with a diagnosis of PSIS in the neonatal period were also excluded because their growth rate before diagnosis could not be calculated.

### Collected data

Social, demographic, and medical data were extracted from the medical report: sex, parental height, and perinatal history. Signs observed before diagnosis and medical and growth records were noted. Data related to the GHRS clinical and auxological criteria were also extracted. During the neonatal period, these criteria are hypoglycemia, prolonged jaundice, microphallus, or traumatic delivery. In the post-neonatal period, they include severely short stature, defined as a height more than 3 SDS below the mean; height more than 1.5 SDS below the target height; height more than 2 SDS below the mean and a height velocity during the previous year more than 1 SDS below the mean for chronological age, or a decrease in height SDS of more than 0.5 over 1 year in children older than 2 years; in the absence of short stature, a height velocity more than 2 SDS below the mean over 1 year or more than −1.5 SD over 2 years [16].

### Definitions

Target height was calculated from parental height [41] and expressed in SDS. Microphallus was defined as a penis length of 2.5 cm or less (−2 SDS) [12]. Height, weight, body mass index (**BMI**, weight in kg/height in m^2^) and height velocity were expressed in SDS for chronological age [31], [42]. Bone age was evaluated by one of us (RB) according to the Greulich and Pyle method [43]. Bone age delay was defined as the difference in years between chronological and bone ages. Thyroid stimulating hormone deficiency was defined by thyroxin level less than 12 pmol/L and adrenocorticotrophin deficiency by basal blood cortisol at 08.00 h less than 70 µg/L.

### Analysis

We first analyzed population characteristics at diagnosis of PSIS with GHD and then studied the medical and growth history, symptoms, and clinical signs through diagnosis. Comparison of each GHRS auxological criterion with growth charts allowed us to establish the age at which each criterion was met, to class each criterion in chronological order of fulfillment, and then to evaluate the diagnostic delay, defined as the difference between the age at which the earliest GHRS criterion was met and the age at diagnosis of PSIS with GHD. We arbitrarily considered a diagnostic delay of one year or more as late diagnosis. Finally, we analyzed each GHRS criterion for how early and with what frequency it was met and arbitrarily defined the most effective criterion as the one that was most sensitive and earliest.
